# Complex Responses of Intertidal Molluscan Embryos to a Warming and Acidifying Ocean in the Presence of UV Radiation

**DOI:** 10.1371/journal.pone.0055939

**Published:** 2013-02-06

**Authors:** Andrew R. Davis, Daniel Coleman, Allison Broad, Maria Byrne, Symon A. Dworjanyn, Rachel Przeslawski

**Affiliations:** 1 Institute for Conservation Biology and Environmental Management, School of Biological Sciences, University of Wollongong, Wollongong, New South Wales, Australia; 2 Schools of Medical and Biological Sciences, University of Sydney, Sydney, New South Wales, Australia; 3 National Marine Science Centre, Southern Cross University, Coffs Harbour, New South Wales, Australia; University of Gothenburg, Sweden

## Abstract

Climate change and ocean acidification will expose marine organisms to synchronous multiple stressors, with early life stages being potentially most vulnerable to changing environmental conditions. We simultaneously exposed encapsulated molluscan embryos to three abiotic stressors—acidified conditions, elevated temperate, and solar UV radiation in large outdoor water tables in a multifactorial design. Solar UV radiation was modified with plastic filters, while levels of the other factors reflected IPCC predictions for near-future change. We quantified mortality and the rate of embryonic development for a mid-shore littorinid, *Bembicium nanum*, and low-shore opisthobranch, *Dolabrifera brazieri*. Outcomes were consistent for these model species with embryos faring significantly better at 26°C than 22°C. Mortality sharply increased at the lowest temperature (22°C) and lowest pH (7.6) examined, producing a significant interaction. Under these conditions mortality approached 100% for each species, representing a 2- to 4-fold increase in mortality relative to warm (26°C) non-acidified conditions. Predictably, development was more rapid at the highest temperature but this again interacted with acidified conditions. Development was slowed under acidified conditions at the lowest temperature. The presence of UV radiation had minimal impact on the outcomes, only slowing development for the littorinid and not interacting with the other factors. Our findings suggest that a warming ocean, at least to a threshold, may compensate for the effects of decreasing pH for some species. It also appears that stressors will interact in complex and unpredictable ways in a changing climate.

## Introduction

Our oceans are changing at an unprecedented rate. Between now and 2100, temperatures are estimated to rise between 2.5 and 4°C [1]. Further, elevated *p*CO_2_ levels from anthropogenic emissions will cause a decline in ocean pH by up to 0.5 units by 2100 [2]; a level of change not observed for 650,000 years [3]. In addition, high latitudes will continue to receive elevated UV radiation for several decades [4]. Evidence is mounting that these potential stressors will have dramatic consequences for both calcifying and non-calcifying marine species [5]–[7], including negative effects on mortality, developmental rate, growth rate and fecundity [8], [9]. However, the magnitude, significance, and even direction of these effects are complex and species-specific [10], [11], with some potential stressors even shown to have positive impacts (e.g. acidification increasing growth rate in non-feeding echinoderm larvae [12]).

Considerable effort is now being expended on predicting how organisms will respond to these challenges, and there is a growing appreciation that abiotic stressors have the potential to interact. Indeed, predicting the responses of organisms to a suite of real-world stressors cannot be achieved with experiments which focus on stressors in isolation [13], [14]. Synergies among climate change stressors have been observed in calcifying algae, with reductions in growth, photosynthetic O_2_ evolution and rates of calcification [15]. Among invertebrates, compensatory effects of temperature on rates of larval calcification in acidified conditions have also been observed [16] although impacts can be strongly species-specific [17].

Responses of organisms to a changing climate are also likely to vary with life history stage; with early life history stages generally considered more vulnerable to stressors than adults [18]. For example, larvae of three species of bivalve were found to be markedly more vulnerable to elevated temperatures and CO_2_ levels than juveniles [11]. However, recent work with ecologically important marine species in SE Australia reveals robust responses of external fertilization to acidified conditions [19], [20], while juveniles were particularly sensitive to climate-related stressors [17], suggesting that gametes and fertilization in many invertebrates exhibit a broad tolerance to warming and acidification [5]. In contrast, fertilisation rate of the Sydney rock oyster was significantly decreased during conditions associated with ocean acidification and warming [21], and elevated temperature and CO_2_ caused deleterious effects across all early life stages of two oyster species, including gametes, embryos, larvae, and spat [22]. These potentially species-specific results highlight an urgent need to better understand how marine species, particularly early life phases, will respond to climate change. The simultaneous exposure of organisms to multiple stressors will ensure that experiments are as realistic as possible.

We investigated the interactive effects of solar UV radiation, pH and temperature on embryonic survival and development for two common intertidal SE Australian molluscs; *Bembicium nanum* (Lamarck) and *Dolabrifera brazieri* Sowerby. Both species spawn year round and consequently are exposed to a wide range of environmental conditions [23]. Molluscan embryos and larvae are known to be vulnerable to ocean acidification through decreases in shell growth and hatching rates [24], [25], and many rocky shore molluscs in SE Australia produce gelatinous or capsular egg masses [26] that are excellent models with which to examine the effects of stressors on early life stages (reviewed by [27]). For example, encapsulated gastropod embryos have been used to investigate the effects of changing environmental conditions on embryonic mortality [28], developmental rate [29], chemical response [30], and cellular response [31]. We predicted that exposing recently laid egg masses to physiologically challenging levels of these stressors would elevate embryonic mortality and prolong development. We also predicted differences in the vulnerability of our test species, as *B. nanum* lays egg masses in shallow pools in the upper mid-shore while *D. brazieri* lays egg masses on the underside of boulders in large low-shore pools. The mid-shore species (*B. nanum*) should be hardier as has been observed previously with a suite of stressors [29], [32]. To our knowledge, this is the first time that early life stages have been investigated in relation to stressors associated with climate change (i.e. temperature, acidification) in conjunction with natural intertidal stressors (i.e. UV radiation). Outcomes will help further inform the design of climate change studies to facilitate more accurate predictions of organism response to changing environmental conditions.

## Materials and Methods

### Study Species and Collection

All necessary permits were obtained for the described field studies. Ethics approval was not required. Collections of invertebrate egg masses were made under New South Wales Department of Primary Industries Scientific Collection Permit F95/269-7.2. Egg masses of the periwinkle snail *Bembicium nanum* (Littorinidae) and the sea hare *Dolabifera brazieri* (Aplysiidae) were collected in the Illawarra region on the New South Wales south coast, Australia (North Wollongong reef: 34°35′45″S, 150°53′20″E; Bellambi reef: 34°37″08″S, 150°92′03″E). These common intertidal molluscs are Australasian endemics and have broad distributions in sub-tropical and temperate Australia [33], [34]. Both species spawn through out the year with summer peaks in egg mass production [23]. Recently spawned egg masses of *B. nanum* were carefully removed from shallow rock pools at the upper mid-shore level. These egg masses are gelatinous and contain eggs measuring 175 µm at a density of approximately 20 eggs mm^−3^ [29]. Egg masses of *D. brazieri* were collected from beneath boulders in the low shore; they consist of thin gelatinous ribbons and contain eggs measuring 75 µm at a density of approximately 66 eggs mm^−3^ [29]. Photographs of these species and their egg masses are presented in [29]. Egg masses of each species were collected on the low tide of the day of experimentation. Only egg masses that were recently laid (ie containing pre-trochophores) were collected, and this was confirmed with a microscope once in the laboratory. Development in these species is rapid; within two days both species develop from yolky pre-trochophores to early stage swimming veligers at moderate temperatures (22°C) ([35] and authors' pers. obs.).

### Manipulative Experiment

We applied the three environmental stressors – solar UV radiation, elevated temperature and acidified conditions, to the developing embryos in a fully orthogonal design. We used three light treatments (full spectrum, UVB blocked and dark); two temperatures (22°C and 26°C) and two *p*CO_2_/pH levels (pH 7.6 and 8.2). This represented the simultaneous application of twelve treatments of these abiotic variables to egg masses. The *p*CO_2_/pH treatment was based on IPCC predictions for 2100, while temperatures were related to predictions of [36] (CSIRO model 3.5) and coincided with average and elevated summer temperatures of rock pools during low tide in this region [29], [37]. Previous work [Figure 1 in [23]) records a maximum water temperature of 23.6°C and a mean maximum air temperature of 26.3°C over a 2 year period for this region. However, temperatures exceeding 35°C in small pools at the upper mid-shore level have also been observed [23]. Experiments were done in large outdoor recirculating seawater tables (3×1.5×0.25 m) so that embryos were exposed to natural light, with largely cloud-free skies. Experiments were conducted during the austral summer in February 2010. This month coincides with an average modelled UV index of 10, just below the peak monthly UV index of 11 in December and January [38]. *Bembicium nanum* and *D. brazieri* were exposed to the same treatments but in separate experiments several days apart; this precluded statistical comparison between the species.

**Figure 1:**
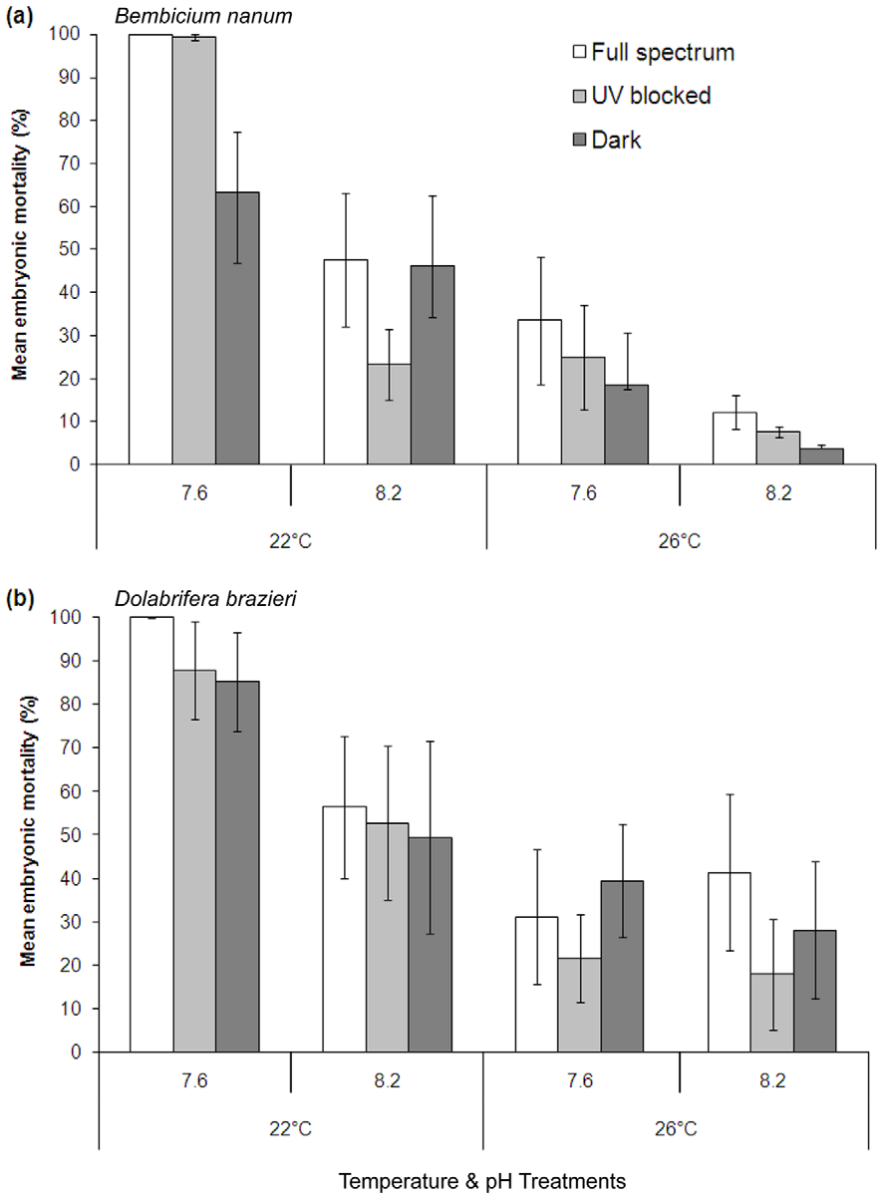
Percent mortality for embryos on exposure to stressors. Effects of temperature, pH, and spectral treatments on embryonic mortality of a) *Bembicium nanum* (n = 8) and b) *Dolabrifera brazieri* (n = 6). Error bars are standard error of mean.

We collected eight egg masses of *B. nanum* and six of *D. brazieri*, reflecting their availability on the days of experimentation. Each egg mass was divided into 12 equal-sized portions and split between each of the 12 treatments; one piece of egg mass per replicate container. By assigning portions of a single clutch (egg mass) to every treatment combination we were able to consider egg mass as a factor in our analysis and in so doing control for inherent genotypic and phenotypic variation. Previous experiments have demonstrated that the division of egg masses into pieces of a similar size does not significantly affect embryonic mortality, but may increase developmental rate [32]. We therefore ensured all egg mass pieces were of equal size so that any increase in developmental rate associated with division would be similar among treatments. Egg mass portions were placed in 120 ml shallow plastic containers (82 mm diameter, 40 mm height). To ensure the flow of oxygenated water through these containers two opposing windows (40×30 mm) were cut from the containers and plastic mesh (1×1 mm) was glued into place (hot melt glue) to ensure the egg masses would remain in the containers. Containers were soaked in seawater for a few days prior to experiments to allow any chemicals associated with the glue and plastics to leach out.

Spectral treatments were achieved with cut-off filters. The top of all containers was covered with clear polyethylene film (Glad™ plastic food wrap) which allowed penetration of full spectrum light and ensured that all spectral treatments came in contact with this material. Solar UV block and dark treatments received additional films; UV was blocked with UV-absorbing polyethylene film, while dark plastic was placed over the top of dark treatments (see [28] for spectral properties of these treatments). UV-A and UV-B were measured regularly with a dosimeter (Grobel RM21). During the duration of the experiments, mean (±SD) UV-A irradiance at solar noon was 6.1 (0.5) W m^−2^ and UV-B irradiance was 0.3 (0.04) W m^−2^. Containers were then positioned haphazardly in one of four 500 L re-circulating seawater tables; two at 22°C and two at 26°C. Temperature was maintained with immersion coolers (Thermoline TIC-400W) and 100–300W aquarium heaters in the sump of each recirculating system. Each pair of temperature controlled baths had two pH levels, 7.6 and 8.2, maintained over the experimental period. Seawater at a pH of 8.2 was collected from the ocean and recirculated while a pH/CO_2_ Controller (TUNZE © model 7074/2) maintained a pH of 7.6 by bubbling CO_2_ gas into the sump of the water table. Air was also bubbled into the sump of each water table to ensure that DO levels were maintained. We added distilled water to the water tables where necessary to ensure that salinity levels remained at 35‰. Temperature and pH_(*NBS*)_ remained relatively constant through out the duration of the 72 hour experiment (TPS™ Aqua pH monitor and Ionode™ Intermediate Junction pH electrode). Due to logistical constraints, replication of water tables was not possible, but frequent monitoring of salinity, water flow, and DO ensured that conditions among all water tables were very similar. All water tables were constructed from fibreglass and had not been used for anything but the circulation of seawater since their manufacture. Water samples were collected at commencement and termination of the experiments and preserved by the addition of saturated mercuric chloride. Total alkalinity of the water samples was determined by potentimetric titrations using the gran calculation method. Aragonite (*Ω*_ar_) and calcite (*Ω*_ca_) saturation levels and the *p*CO_2_ in the treatments were calculated using CO2SYS [39].

### Environmental stressors and development

Seventy two hours after initiating the experiments we examined egg masses with dissecting microscopes (×40 magnification), determining percent mortality and the developmental stage following the methods of [32]. Mortality was estimated for each portion of egg mass by scoring 100 embryos as dead or alive, with degenerating or relatively underdeveloped embryos considered dead. For each portion of egg mass, developmental stage was recorded according to the stage in which the majority of embryos were found. If two stages were equally represented within one piece, the mean stage was recorded. Developmental stages were classified as (1) nonciliated (pre-trochophore or undeveloped eggs), (2) ciliated with no shell (trochophore/early veliger), (3) partial shell (veliger), and (4) full shell (late veliger/larva).

### Statistical Analyses

We used restricted maximum likelihood ANOVAs to determine treatment effects for each species; the random factor was the individual egg mass from which portions were obtained. Mortality data showed a binomial distribution and were arcsin square root transformed to satisfy ANOVA assumptions of a normal distribution. For all significant factors and interactions, Tukey's HSD tests revealed significant *a posteriori* relationships between treatments (see Table S1). Analyses were performed in JMP v. 8 with α = 0.05.

## Results

### Mortality

Temperature and pH stressors were key drivers of embryonic mortality for both mollusc species (Figure 1), while spectral treatment had no significant effect. Mortality was elevated at the lower temperature (22°), particularly in acidified water (pH 7.6) for both species; often approaching 100% (Figure 1). In contrast, mortality was usually less than 25% for both species in warm (26°C) non-acidified water. Our analyses confirmed the significant interaction between temperature and pH (*B. nanum P* = 0.0263, *D. brazieri P* = 0.0471, Table 1); no other interactions were significant.

**Table 1:** The effects of temperature, pH, and spectral treatment on embryonic mortality (arcsin transformed) as determined by ANOVAs using restricted maximum likelihood with random factor italicized.

	*Bembicium nanum* (n = 8)			*Dolabrifera brazieri* (n = 6)		
Factor	df	*F*	*P* 1	df	*F*	*P* 1
*Egg mass*	7	na	0.6201–0.9108	5	na	0.1809–0.6114
pH	1	23.4824	<0.0001	1	7.3340	0.0090
Temperature (T)	1	60.7523	<0.0001	1	18.0012	<0.0001
Spectral (UV)	2	2.3635	0.1009	2	0.6623	0.5197
pH×UV	2	1.2333	0.2970	2	0.3399	0.7133
pH ×T	1	5.1344	0.0263	1	4.1264	0.0471
T ×UV	2	0.1382	0.8712	2	0.6399	0.5312
pH×UV×T	2	1.5517	0.2184	2	0.1838	0.8326

See Table S1 for outcomes of *a-posteriori* comparisons (Tukey's HSD).

1 P-values for random factors are based on results of t-tests using Best Linear Unbiased Predictors, parameter estimates associated with random effects in the REML model performed in JMP v. 8.

### Developmental Stage

**Figure 2:**
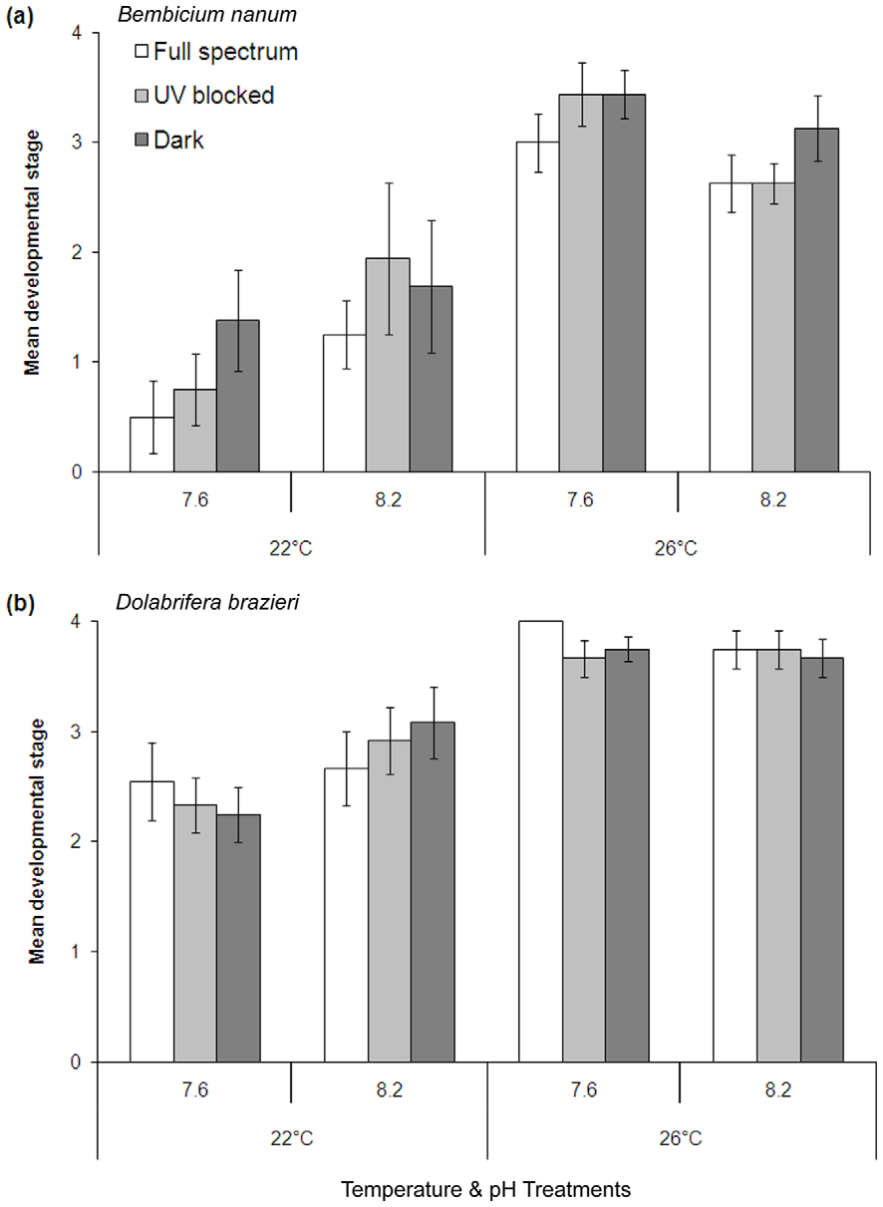
Mean developmental rate for embryos on exposure to stressors. Effects of temperature, pH, and spectral treatments on developmental rate of a) *Bembicium nanum* (n = 8) and b) *Dolabrifera brazieri* (n = 6). Developmental stages were classified as (1) nonciliated (pre-trochophore or undeveloped eggs), (2) ciliated with no shell (trochophore/early veliger), (3) partial shell (veliger), and (4) full shell (late veliger/larva). Error bars are standard error of mean.

As expected, development was significantly faster at 26°C than 22°C for both species (Figure 2). There was also evidence of an elevation in development rate of *B. nanum* at pH 7.6 in warm water (26°C), although this trend was reversed at 22°C. Our analyses confirmed a significant interaction between temperature and pH on the rate of development in both species (*B. nanum P* = 0.0007, *D. brazieri P* = 0.0169, Table 2). Again, this was the only interaction that was significant. Full spectrum light (which includes UV radiation) consistently slowed development across all temperature and pH combinations for *B. nanum* (*P* = 0.0125) relative to UV block and dark treatments. No consistent trend in spectral treatment was apparent for the developmental rate of *D. brazieri* (*P* = 0.8720).

**Table 2:** The effects of temperature, pH, and spectral treatment on developmental rate as determined by ANOVAs using restricted maximum likelihood with random factor italicized.

	*Bembicium nanum* (n = 8)			*Dolabrifera brazieri* (n = 6)		
Factor	df	*F*	*P* 1	df	*F*	*P* 1
*Egg mass*	7	na	0.0720–0.4178	5	na	0.0551–0.3726
pH	1	0.5049	0.4795	1	3.1435	0.0818
Temperature (T)	1	154.6311	<0.0001	1	87.8266	<0.0001
Spectral (UV)	2	4.6389	0.0125	2	0.1373	0.8720
pH×UV	2	0.0316	0.9689	2	1.3621	0.2646
pH×T	1	12.6230	0.0007	1	6.0702	0.0169
T×UV	2	0.0316	0.9689	2	0.3282	0.7216
pH×UV×T	2	2.1143	0.1277	2	0.4713	0.6267

See Table S1 for outcomes of *a-posteriori* comparisons (Tukey's HSD).

1 P-values for random factors are based on results of t-tests using Best Linear Unbiased Predictors, parameter estimates associated with random effects in the REML model performed in JMP v. 8.

## Discussion

Our data confirm that an acidifying ocean can interact with other climate change stressors to produce complex patterns of mortality and modify developmental rate. Overall, acidification lowered survivorship and developmental rate at moderate temperatures, but these effects seemed to be mitigated by warmer temperatures. Our initial predictions were not accurate, as the most physiologically demanding conditions (full spectrum, pH 7.6, 26°C) did not produce the highest mortality nor significantly slow development.

Results from this study and others do not reveal a clear pattern regarding the potentially interactive effects of pH with other stressors on larval development. We found a significant interaction between pH and temperature on the development of both species but not in the direction predicted. Specifically, pH decreased survival and developmental rates but only at the lower temperature we tested. In contrast [40], reported significant individual effects of temperature and pH on the development of a non-calcifying seastar but did not detect any interactions. Other workers [11] have reported that elevated temperature and CO_2_ significantly interacted to affect some developmental metrics of bivalves, but the significance and magnitude of these interactions varied among species and the metrics used (survival, developmental rate, growth, lipid synthesis). Such differences were also detected in experiments on the Sydney rock oyster in which elevated temperature and CO_2_ interacted significantly to affect growth, abnormality, and mortality rates, but only individual factors affected fertilisation success [21]. These complex effects may be attributed in part to differences in physiological mechanisms, particularly among species (e.g. acid-base regulatory capacity [10]).

Irrespective of spectral or pH treatments, embryos fared significantly better at 26°C than 22°C. The impacts of temperature on developing embryos and larvae are well known to developmental biologists, and high temperatures have been identified as the primary stressor in some climate change and ocean acidification experiments [19], [40]. It has been argued that thermal windows may in fact determine how species will respond to a changing climate, with the presence of other non-thermal stressors narrowing these windows [41]. It should be remembered though that thermal affects might go beyond physiological responses and modify the risks to which organisms are exposed. For example, elevated temperatures will reduce the period of encapsulation thus reducing exposure to sources of mortality, such as predators or UV radiation, for highly vulnerable life history stages [42], [43].

Temperature was a key driver of the outcomes in our experiments, particularly at low pH; embryonic survival was poor at the lower temperature we tested. Other researchers have similarly reported the effects of a stressor to be more apparent at lower temperatures. Larval amphibians suffered higher mortality, lower growth and reduced mobility on exposure to UV radiation at low temperatures [44], and the brown alga *Alaria marginata* similarly did not survive exposure to UV radiation at low temperatures [14]. Elevated temperatures increase developmental rate and may also increase the associated rate at which embryos develop protective mechanisms [27]. For example, several workers have speculated that as enzymatic rates are linked to temperature, photolyase-mediated DNA repair will be more effective at elevated temperatures [29], [45], [46].

Under conditions of climate change, increasing temperatures may, at least to a threshold, counteract the negative effects of other abiotic stressors. Importantly, potential thermal thresholds for our model species and their egg masses remain unknown, and at temperatures higher than 26°C, the negative effects of pH may be even more pronounced. The use of an additional thermal treatment (eg 30°C) would have been instructive in this regard. We also note that the egg masses faring better in our experiments at 26°C are unlikely to reflect acclimation to high temperatures as our collection of egg masses equated to a relatively cool summer in SE Australia with an average maximum air temperature of just 23.2±0.7°C (±SD) for the first 10 days of February 2010 [47].

Interpretation of the effects of ocean acidification on intertidal organisms must take account of fluctuations in natural pH levels in their environment. It has long been known that significant diurnal fluctuations in *p*CO_2_/pH occur in intertidal pools due to algal metabolism [48], [49]. As such, coastal species such as those used in our experiments may be exposed to rapid pH fluctuations [50], with acidified conditions substantially below the scenarios anticipated by 2100 [1]. Intertidal organisms may therefore be quite resilient to the comparatively slower and less pronounced changes in pH due to ocean acidification [19]. Contrary to this prediction, our study showed that both *B. nanum* and *D. brazieri* were vulnerable to decreased pH. However, we anticipate that the small pools devoid of algae that are favoured by *B. nanum* and the open subtidal habitats that are favoured by *D. brazieri* are less likely to undergo dramatic fluctuations in pH than small rock pools with abundant algae.

Previous studies have focused on the responses of organisms to an acidifying aqueous environment often incorporating elevated temperatures to mimic climate change scenarios (e.g. [14], [19]). As far as we are aware, however, the current study is the first to simultaneously expose organisms to an additional stressor – UV radiation. Elevated UV radiation can greatly increase the negative impacts of other stressors such as toxins (reviewed by [51]), but it played a relatively minor role in our trials; full-spectrum light only proved to slow development in *B. nanum*. This outcome was unexpected, as *D. brazieri* embryos have shown extreme sensitivity to ultraviolet radiation in previous experiments [29], [32]. In contrast, embryos of *B. nanum* have been shown to vary in their response to UV radiation [29], [32], suggesting that the vulnerability of embryonic gastropods to UV radiation and potentially other stressors may vary among egg masses due to individual or cohort variation associated with maternal provisioning or acclimation. Indeed, it would be predicted that organisms inhabiting pools with substantial nocturnal decreases in pH would have higher fertilisation rates when exposed to lower pH than those that inhabit rockpools with stable pH and there appears to be some data in support of this assertion [50]. Similarly, oyster larvae have shown increased resilience to elevated pCO_2_ after paternal exposure of adults, suggesting that adult exposure to stressors can subsequently affect the vulnerability of offspring [52]. Alternatively, variation in the vulnerability of embryos may instead reflect differences among seasons, locations, or other environmental factors we did not consider [53].

Our findings reinforce the importance of inferring climate change effects from multi-factorial experiments – a point that has been emphasised by several authors [5], [14], [32]. It should be noted though that our experiments exposed egg masses to rapid environmental change; more rapid than anticipated under climate change scenarios. Evidence of the capacity of marine organisms to acclimate comes from recent work on thermal tolerances of encapsulated molluscs; low latitude populations were more tolerant to elevated temperatures that those from high latitudes [54]. Although challenging for ecologists, it is now essential to focus climate change research on whole-ecosystem responses to multiple stressors [13], [55]. In addition, an understanding of the physiological processes underlying such responses (e.g. [10] may help identify patterns among marine invertebrates and facilitate predictions of climate change and ocean acidification impacts.

## Supporting Information

Table S1 Outcomes of Tukeys HSD *a posteriori* comparison for significant Temperature×pH interactions.
(DOCX)
