# Supplementary material for: Complex Responses of Intertidal Molluscan Embryos to a Warming and Acidifying Ocean in the Presence of UV Radiation
Source: PLoS One. 2013 Feb 6;8(2):e55939. doi: 10.1371/journal.pone.0055939 (PMC3566103; doi:10.1371/journal.pone.0055939)
Supplement: Table S1 — Outcomes of Tukeys HSD a posteriori comparison for significant Temperature×pH interactions. (DOCX) [file pone.0055939.s001.docx]

**Table S1**

Taxon Ranked sample means

*Bembicium nanum*

Mortality *‾Χ_1_ ‾Χ_2_ ‾Χ_3_ ‾Χ_4_*

Development  *‾Χ_1_ ‾Χ_2_ ‾Χ_4_ ‾Χ_3_*

*Dolabrifera brazeri*

Mortality *‾Χ_1_ ‾Χ_2_ ‾Χ_3_ ‾Χ_4_*

Development  *‾Χ_1_ ‾Χ_2_ ‾Χ_4_ ‾Χ_3_*

*Where:*

*‾Χ_1_ =* 22°C, pH 7.6

*‾Χ_2_ =* 22°C, pH 8.2

*‾Χ_3_ =* 26°C, pH 7.6

*‾Χ_4_ =* 26°C, pH 8.2
